# Genomic landscape of *Candidozyma auris* in Italy, 2019 to 2025: emerging diversity of clade I sub-lineages associated with inter-facility transmission and cross-border transfers

**DOI:** 10.2807/1560-7917.ES.2026.31.18.2500730

**Published:** 2026-05-07

**Authors:** Vincenzo Di Pilato, Edward Willison, Luca Calabrese, Elisa Matarazzo, Laura Magnasco, Elisa Costa, Paola Morici, Daniele Roberto Giacobbe, Chiara Vismara, Malgorzata Mikulska, Gian Maria Rossolini, Matteo Bassetti, Anna Marchese

**Affiliations:** 1 Department of Surgical Sciences and Integrated Diagnostics (DISC), University of Genoa, Genoa, Italy; 2 Microbiology Unit, IRCCS Azienda Ospedaliera Metropolitana, Genoa, Italy; 3 Clinical Microbiology Unit, ASST Grande Ospedale Metropolitano Niguarda, Milano, Italy; 4 Infectious Diseases Unit, IRCCS Azienda Ospedaliera Metropolitana, Genoa, Italy; 5 Department of Health Sciences (DISSAL), University of Genoa, Genoa, Italy; 6 Department of Experimental and Clinical Medicine, University of Florence, Florence, Italy; 7 Microbiology and Virology Unit, Careggi University Hospital, Florence, Italy

**Keywords:** fungal pathogen, epidemiology, genomic surveillance, WGS, infection prevention and control, screening

## Abstract

**BACKGROUND:**

*Candidozyma auris* is a fungal pathogen of major concern, that frequently exhibits multidrug resistance and causes healthcare-related outbreaks worldwide. Italy experienced a large nosocomial outbreak in early 2020, with subsequent sporadic cases or small clusters in different regions.

**AIM:**

To provide an overview of the *C. auris* population structure, genomic diversity, and spread in Italy.

**METHODS:**

Genome sequences from Italian *C. auris* isolates (n = 68) were obtained either from public databases, or by whole-genome sequencing of available isolates (n = 17) from previously uncharacterised and/or recently emerged (2025) cases. The sequence dataset was complemented with whole genome sequences of international isolates (n = 139) to conduct a global phylogenetic analysis based on core-genome single nucleotide polymorphisms. Genetic mutations associated with antifungal resistance were investigated.

**RESULTS:**

All Italian isolates belonged to clade I (South Asian) but were interspersed among different subclades. Subclade Ic isolates were of a single lineage, characterised by the *HMG1*^P238H^ mutation. This lineage spread over four regions. Subclade Ib members were more diverse, and associated with local or imported single cases, as well as nosocomial clusters following sporadic, independent *C. auris* introductions from countries in southern Europe. A putative new subclade (Id) was identified, involving isolates from Italy and an eastern European country. Subclades Ib-c-d exhibited lineage-specific genetic signatures in antifungal-resistance-associated loci (*CDR1, ERG11, TAC1B*).

**CONCLUSIONS:**

In Italy, *C. auris* strains form a complex population, resulting from emergence or evolution of clade I sub-lineages following, in some instances, sporadic introductions from other European countries. Strengthened screening protocols remain essential for inter-facility transfers and for patients with prior healthcare exposure abroad.

**Key public health message**

**What did you want to address in this study and why?**

*Candidozyma auris* is a fungal pathogen that can cause invasive infections and exhibit concerning drug resistance. To inform control strategies, we investigated the diversity and transmission dynamics of *C. auris* in Italy. For this, we retrieved all Italian *C. auris* whole-genome sequences publicly available and obtained new whole-genome sequences from recent/not yet characterised *C. auris* clinical samples. The resulting dataset was analysed in the context of international sequences.

**What have we learnt from this study?**

In Italy, *C. auris* dissemination exclusively involved the phylogenetic clade I (South Asian origin). The pathogen spread was largely linked to a major national outbreak starting in 2020, with interregional transmissions. Genomic analyses revealed unrelated sporadic cases or clusters of cases in multiple national hospitals, likely driven by *C. auris* introductions from countries in south-eastern Europe. Some imported cases indirectly suggested *C. auris* circulation in so far unsuspected European areas.

**What are the implications of your findings for public health?**

The present findings support the strengthening of infection prevention and control measures, including systematic screening of patients transferred between Italian hospitals, and patients with recent healthcare exposures abroad, notably from countries where information on *C. auris* circulation is scarce or lacking. Genomic surveillance is critical to detect new introductions, monitor transmission, and guide targeted interventions to contain spread of *C. auris* in hospitalised patients.

## Introduction

Since it was first described in 2009, the yeast *Candidozyma* (*Candida*) *auris* has been causing challenging nosocomial outbreaks of invasive infections worldwide [1]. Due to this and the detection of strains with concerning drug resistance profiles, the World Health Organization (WHO) categorised this species as a critical fungal pathogen in 2022 [1]. Moreover, in just over 16 years following its identification, *C. auris* epidemiology in several countries has evolved, with outbreaks of healthcare-associated infections giving way to endemic transmission [1]. Additionally, pandrug-resistant isolates have been reported, particularly in settings with sustained circulation of *C. auris*, including in the United Kingdom (UK; 2018) [2], the United States (US; 2019) [3], India (2017–2020) [4], Spain (2019–2020) [5], Greece (2022) [6], and Italy (2022) [7].

Genomic-informed studies have revealed that the global spread of *C. auris* is driven by the simultaneous emergence of distinct phylogenetic clades in various geographical areas of the world. To date, six clades (I to VI) have been described, and typing by whole genome sequencing (WGS) has proven instrumental to track *C. auris* dissemination in hospitals and community settings [8].

In the European Union/European Economic Area (EU/EEA), the European Centre for Disease Prevention and Control (ECDC) reported in September 2025, a total of 4,012 cases of *C. auris* colonisations or infections between 2013 and 2024 [9]. During this period, there was a near doubling of the number of cases from 2022 to 2023 [9], and, coinciding with the COVID-19 pandemic, considerable shifts in the epidemiological trends of bloodstream infections were observed in Greece (2021–2023) [10] and Spain (2019–2021) [11], where *C. auris* progressively emerged as a prominent causative agent of candidaemia. At present, Spain, Greece, Romania, and Italy have the highest *C. auris* case numbers in the EU/EEA, with circulating strains predominantly belonging to clades I and III [9,12], and to a lesser extent to clade IV [13]. Several cases have also been reported in EU enlargement countries, such as some in the Western Balkans and Türkiye [9].

Notification of carriage and infection cases of *C. auris* in Italy has been required since June 2020, when the Italian Ministry of Health issued a Circular Letter (number 0019275–05/06/2020-DGPRE-DGPRE-P) providing information for prompt identification and management of such cases. The emergence and spread of *C. auris* in the country has been primarily associated with a large outbreak recognised in early 2020 in a single region [14]. Nevertheless, sporadic cases or small clusters have been subsequently identified at national level [15], contributing to a progressive increase in case numbers throughout 2020–2025. According to the latest Circular Letter issued by the Italian Ministry of Health (number 0004265–12/02/2025-DGPRE-DGPRE-P, February 2025), *C. auris* has been detected in several regions during 2023 up to August 2024, raising concerns about an increased risk of intrahospital transmission.

Although continuous efforts have been made to conduct genomic investigations in Italy, these have primarily focused on local transmission events, with a lack of in-depth comparative analyses at larger geographical scale. Here, we more widely explored the genomic diversity and transmission dynamics of *C. auris*, by analysing whole genome sequences from across the country, in the context of international sequences. 

## Methods

### Study design

To perform comprehensive comparative analyses, we established a dataset with *C. auris* nucleotide sequences respectively derived from Italian isolates. This was done by retrieving raw reads or genome assembly data related to studies indexed by PubMed (accessed on 15 January 2026) using the following search queries without date or language restrictions: *((Candidozyma) AND (auris)) AND (Italy); ((Candida auris) AND (Italy)) AND (identification); ((Candida auris) AND (Italy)) AND (emergence); ((C. auris) AND (Italy)) AND (Italian)*) and from the National Center for Biotechnology Information (NCBI) genome and Sequence Read Archive (SRA) databases (https://www.ncbi.nlm.nih.gov/sra/?term=candida+auris, accessed on 15 January 2026) through the RunSelecter tool. A total of 68 genome records were selected, including genome sequences from the Italian major outbreak in Liguria (n = 60, July 2019–October 2022) [7,14], and genome sequences from sporadic cases reported in Emilia Romagna (n = 3) [16], Apulia (n = 3) [17], Lombardy (n = 1) [18], and Marche (n = 1) [19]. Typing by WGS of available, previously uncharacterised and/or recently emerged cases of *C. auris* (both surveillance and clinical samples) from Lombardy (n = 15, September 2022–March 2025), Liguria (n = 1, August 2023), and Tuscany (n = 1, March 2025) was performed as detailed below. The final dataset consisted of a total of 85 genome sequences of *C. auris* isolates from Italy (i.e. 68 publicly available genome records plus 17 genomes generated in this study). Epidemiological data (e.g. prior exposure to healthcare abroad, patient transfer, spread) were obtained from previously published reports [7,14-24], and retrospectively investigated for uncharacterised and/or recently emerged cases of *C. auris* (i.e. originating from Lombardy, hospital B, and Tuscany).

### Storage of fungal isolates and culturing conditions

All fungal isolates were stored at − 80 °C in Brain Heart Infusion (BHI) broth (Oxoid, Hampshire, UK) containing 20% v/v glycerol (Sigma Aldrich, Saint-Louis, Missouri, US). The glycerol stocks were used as sources of isolates for all the experiments performed in this study. From frozen stocks, isolates were plated on Sabouraud Dextrose plates (Oxoid) and incubated overnight at 37 °C before downstream analyses (i.e. genomic DNA extraction, antifungal susceptibility testing).

### Whole genome sequencing and bioinformatics

Total genomic DNA was extracted from fungal cultures grown in YPD broth (Oxoid), using the Qiagen DNeasy PowerLyzer PowerSoil Kit (Qiagen, Hilden, Germany). Shotgun libraries were prepared from purified genomic DNA samples and sequenced with the NovaSeq platforms (Illumina, San Diego, CA), using a 2 × 150 bp paired-end approach. Raw reads were assembled using SKESA v2.4.0 (https://github.com/ncbi/SKESA). Global phylogenomic investigations were based on core-genome single nucleotide polymorphisms (SNPs). To uncover these, BWA v0.7.17 was used to align the Illumina reads against the last revision of the *C. auris* B8441 reference genome from clade I (GenBank assembly accession number: GCA_002759435.3) [25], and NUCmer v3.1 and FreeBayes v1.1.0 were employed to identify repetitive regions and call SNPs, respectively [26,27]. Maximum-likelihood phylogenetic trees were constructed based on concatenated core-genome SNPs using IQ-TREE v1.6.12, using a general time reversible (GTR) nucleotide substitution model [28].

The genome sequences of international isolates (n = 112) from validated outbreak benchmark datasets were employed in the global analysis, for clade and subclade assignation [29,30]; these included some sourced from outbreak clusters previously described in healthcare facilities in New York, New Jersey, and Massachusetts [29]. Sequences of most recent clade VI isolates [31] as well as of *C. auris* strains from Romania (n = 27) were also used in the analysis to provide additional genetic context [32]. Altogether, they originated from 17 countries comprising Canada, Colombia, India, Iran, Israel, Japan, Kenya, Pakistan, Romania, Saudi Arabia, Singapore, South Africa, South Korea, United Arab Emirates, UK, US and Venezuela. A complete list of genome records analysed in this study along with associated metadata are available in Supplementary Table S1.

The genome sequence of the *C. auris* strain B8441 (GenBank assembly accession number: GCA_002759435.3) was used as reference to verify the presence of mutations possibly involved in antifungal resistance. Screening for mutated alleles within the global *C. auris* genome dataset was performed using k-mer alignment (KMA) [33].

## Results

### Spread of *Candidozyma auris* in Italy

**Figure 1:**
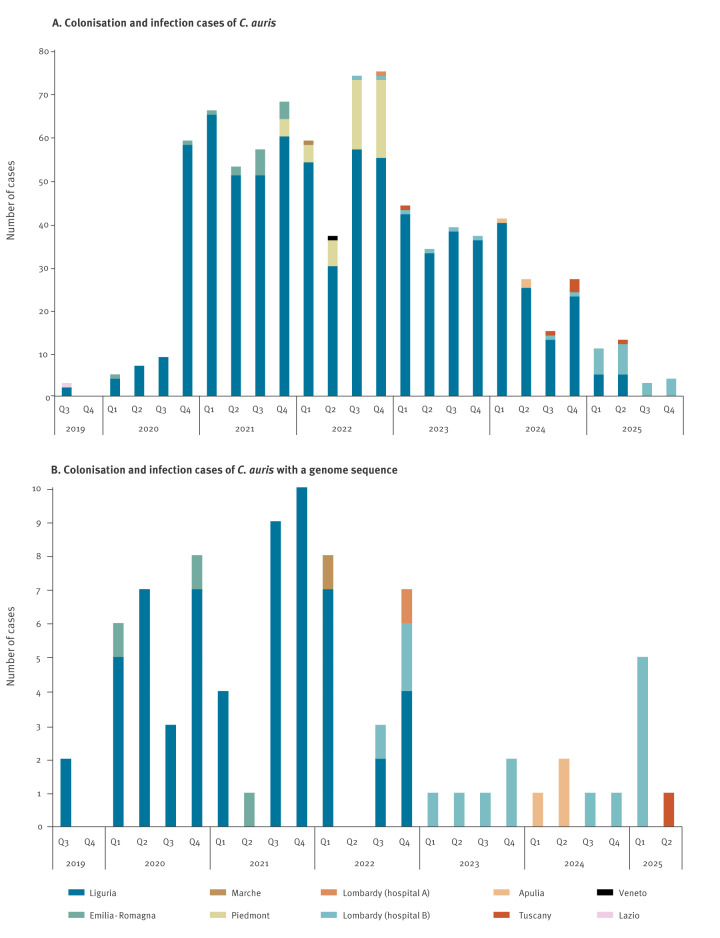
Temporal and regional distribution of (A) *Candidozyma auris* colonisation and infection cases (n = 867)^a^, and of (B) a subset of cases, for which a *C. auris* genome was available (n = 85), Italy, 2019–2025

^a^ The 867 cases included some identified through previously published data [7,14-19,21-24] and some newly reported in the current study.

In Italy, *C. auris* was first identified in July 2019, in a large tertiary care hospital in northern Italy (Liguria region), upon diagnosis of candidaemia and skin colonisation in a patient with no documented previous healthcare exposure [14]. Thereafter, from early 2020, a sharp rise in the number of carriage and infection cases was observed at the same hospital. This led to a difficult-to-control outbreak, which was likely facilitated by the ongoing COVID-19 pandemic [3], with 763 cases recognised by the end of 2025 (this study) (Figure 1A). Meanwhile, the outbreak spread further in the same region, where at least eight healthcare facilities were affected by *C. auris* by February 2022 [20]. Since mid-2024, however, a marked decline in total cases has been observed at the primary hospital affected in Liguria, with only sporadic cases reported in the first half of 2025 and no new cases detected throughout the second half of that year (Figure 1A); taken together, these findings may indicate that the outbreak in this hospital may be in the process of being resolved.

At the national level, sporadic cases were progressively described also in other regions (Figure 1A), including Piedmont (n = 48, December 2021–December 2022 [15]), Emilia-Romagna (n = 15, January 2020–December 2021 [15]), Lazio (n = 1, November 2019 [21]), Veneto (n = 1, June 2022 [22]), Marche (n = 1, January 2022 [19]), Lombardy (hospital A, n = 1, October 2022; hospital B, n = 28, September 2022–December 2025 [18]), Apulia (n = 3, April 2024 [17]), Tuscany (n = 6, March 2023–March 2025 [23,24] and this study).

**Figure 2:**
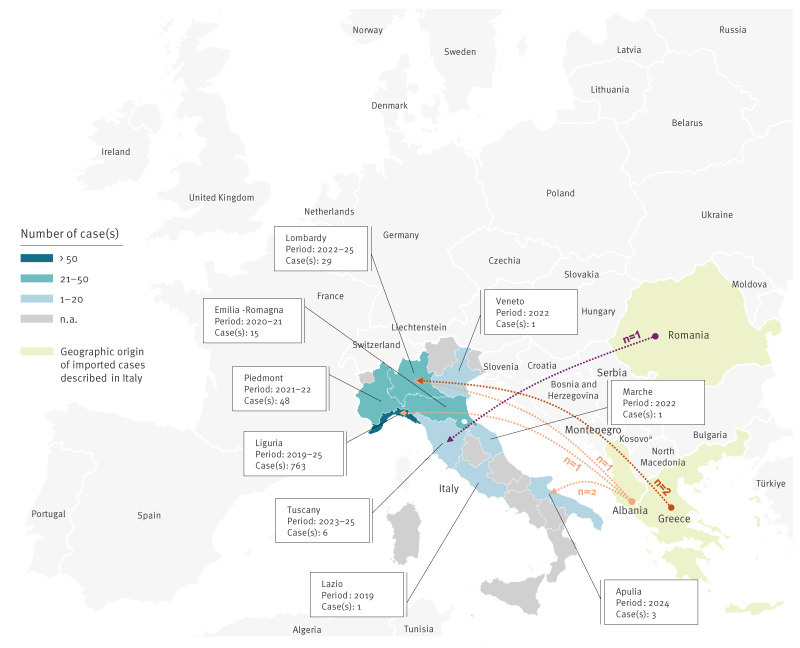
Overview of *Candidozyma auris* cases and spread in Italy, with possible importations of four cases from neighbouring southern European countries, 2019–2025 (n = 867 cases)

A minority of cases were patients who had a documented history of hospitalisation abroad, including Greece (n = 2), Romania (n = 1) and Albania (n = 4), suggesting that these could be imported cases (Figure 2). However, detailed epidemiological links were not always known, as *C. auris* cases have been inconsistently epidemiologically investigated to date, and genomic information of the pathogen population structure and clonal relatedness remains partial.

n.a.: not applicable.

^a^ This designation is without prejudice to positions on status and is in line with UNSCR 1244 and the IСJ Opinion on the Kosovo Declaration of Independence.

Italian regions affected by *C. auris* are shaded in various intensities according to their number of cases of infection/colonisation. Details about the period when *C. auris* was isolated are provided for each region. Countries outside Italy, which were prior visited by seven possible imported cases are highlighted in light green, with dotted lines of different colours linking the prior visited countries with the respective Italian regions where the cases were detected.

### Genomic census and population structure of *Candidozyma auris* in Italy

Building on previous studies, we retrieved all publicly available WGS data of *C. auris* isolates from Italy, including data from the major outbreak in Liguria (n = 60) and from national cases reported in Emilia-Romagna (n = 3), Apulia (n = 3), Lombardy (hospital A, n = 1), and Marche (n = 1) (Figure 1B). We also sequenced the whole genome of available isolates that had previously not been characterised and/or from recently emerged cases in Lombardy (hospital B, n = 15, September 2022−March 2025), Liguria (n = 1, August 2023), and Tuscany (n = 1, March 2025) (Figure 1B). A comprehensive phylogenetic analysis was then conducted on WGS data of *C. auris* isolates (n = 85) collected in Italy over the July 2019−March 2025 period and WGS data of international isolates (n = 139) sourced from validated benchmark datasets for clade and subclade assignation [29,30,32], including most recent isolates from clade VI [31]. A complete list of analysed isolates, and associated metadata, is available in Supplementary Table S1.

**Figure 3:**
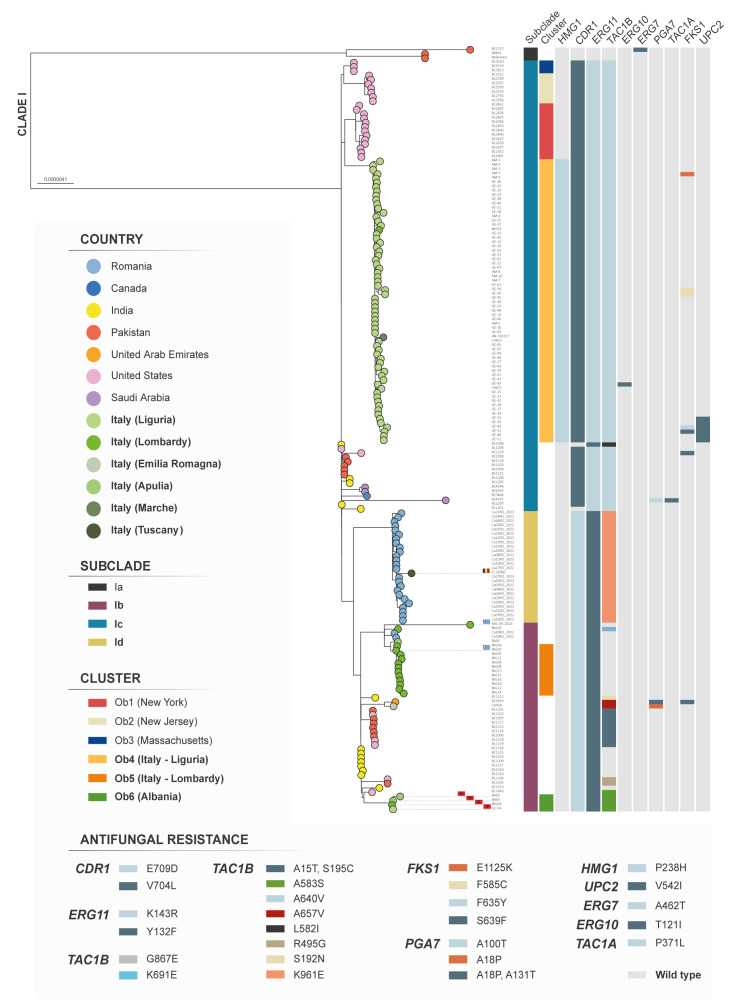
Analyses of *Candidozyma auris* sequences from Italy, 2019–2025 (n = 85), for mutations involved in antifungal resistance and by maximum likelihood phylogeny, using benchmark datasets including clade I (South Asian) sequences^a^ and sequences from Romania^a^ (n = 27)

^a^ The dataset with benchmark sequences from clade I (South Asian) [29,30], as well as the sequences from Romania [32] have been previously reported.

Results showed that in agreement with previous reports [7,16,17], all Italian isolates belonged to clade I (South Asian), as illustrated in Supplementary Figure S1. Nevertheless, phylogenetic analysis showed heterogeneity in the population structure, as also suggested by the wide range of SNPs between the isolates’ sequences (min: 0, max: 208; median: 13; interquartile range (IQR): 6–102) (Figure 3). While none of the Italian sequences were assigned to subclade Ia, they distributed among several other subclades, in the same way than sequences from India, Pakistan, and the US (Figure 3). 

The scale bar on the tree indicates the number of nucleotide substitutions per site. On the tree leaves, the colours of the isolate tips represent the country of isolation; the national flag of the source country is reported next to the tree tip of deemed imported cases. In the figure legend, the text reported in bold font refers to sequences identified in Italy, with the texts in parentheses indicating the likely origin of *C. auris* (Italian region or foreign country). Details about subclade designation, and genetic mutations associated with azoles (*CDR1*, *ERG11*, *TAC1B*, *HMG*, *UPC*, *ERG7*, *ERG10*, *PGA7*, *TAC1A*) and echinocandins (*FKS1*) resistance are also provided.

Remarkably, within subclade Ic, all but one Italian isolate (n = 60) from the major outbreak in Liguria formed a monophyletic group. The group additionally included isolates from cases identified in Lombardy (hospital B, 2022, n = 1), Emilia Romagna (2020–2021, n = 2) and Marche (2022, n = 1). This suggests the clonal expansion of a single lineage with inter-regional spread (cluster Ob4, n = 64; Figure 3), a hypothesis also supported by the limited numbers of SNPs between the isolates’ sequences (min: 0, max: 24; median: 7; IQR: 5–11) and by a conserved genetic signature previously associated with the main outbreak occurring in Liguria (i.e. *HMG1*^P238H^) [7]. Aside from the group of Italian sequences in subclade Ic, separate benchmark clusters with sequences from the US could be observed, including cluster Ob1 from New York, Ob2 from New Jersey and Ob3 from Massachusetts (Figure 3) [29,30].

Conversely, the remaining isolates were heterogeneously distributed across subclade Ib (Figure 3). The phylogenetic analysis resolved a paraphyletic group (cluster Ob5, n = 12) comprising isolates from a single hospital in Lombardy (hospital B, 2022–2025, n = 12), showing a high genomic relatedness consistent with clonal expansion of a single lineage (SNPs min: 0, max: 13; median: 5; IQR: 3–7); retrospective epidemiological investigations in the current study revealed that the earliest isolate in this group was recovered from a patient transferred from a Greek hospital (November 2022), suggesting that this introduction may have prompted the subsequent local transmission. Additionally, a monophyletic group (cluster Ob6, n = 4) comprising isolates from different hospitals located in southern Italy (Apulia, 2024, n = 2) and in northern Italy (Liguria, 2023, n = 1; Lombardy, hospital B, 2024, n = 1) was identified. Each of the isolates in this group bore the antifungal resistance marker *TAC1B***^A583S^**, moreover, these four isolates were characterised by a high genomic relatedness (SNPs min: 3, max: 12; median: 5; IQR: 3–11) pointing to a clonal origin. All of them were recovered from patients with recent history of healthcare exposure in Albania, as noted earlier [17] and in this study.

The subclade Ib also included benchmark isolates characterising this subclade (e.g. B13916, B11118, B11119, B11102, B11104, B13343) [30], as well as clonally unrelated isolates (i.e. individual lineages) represented by an imported case from Greece (n = 1; MIL-ITA-2023; Figure 3), previously reported in Lombardy (hospital A, 2022) [18], a case from southern Italy (BA01; Apulia, 2024) with no information about healthcare exposure abroad [17], one additional unrelated case from Lombardy (NIG03) and two recently characterised cases from Romania (Ca19RO, Ca20RO) [32] (Figure 3).

Of note, the case recently reported in Tuscany (2025; FI-32066; Figure 3), which was likely imported from Romania (n = 1), and whose isolate sequence was characterised in this work, clustered in a monophyletic group together with other contemporary isolates (2022–2023, n = 25) reported from different Romanian (Bucharest) hospitals (Figure 3) [32]. All isolates within this cluster (n = 26) shared a distinctive mutation in an antifungal resistance locus (e.g. *TAC1B*^K691E^), as well as a limited number of separating SNPs (min: 0, max: 28; median: 12; IQR: 8–16). According to phylogenetic analysis, this group could possibly define a novel subclade, hereafter referred to as subclade Id. The lack of the *TAC1B*^K691E^ marker in the two Romanian cases clustering in subclade Ib (Ca19RO, Ca20RO; Figure 3), together with their SNPs distance from Romanian cases clustering in the putative subclade Id (min: 90, max: 111; median: 96; IQR: 94–99) and previous findings from Surleac et al. [32], further support this hypothesis.

Analysis of loci commonly involved in antifungal resistance revealed the presence of multiple alterations associated with the population structure at the subclade level, such as *CDR1*^V704L^, *ERG11*^K143R^, *TAC1B*^A640V^ in subclade Ic, *CDR1*^E709D^, *ERG11*^Y132F^ in subclades Ib and Id, and *TAC1B*^K691E^ in subclade Id (Figure 3, Supplementary Table S1).

## Discussion

According to an ECDC report from September 2025, the number of *C. auris* infections and carriage cases in the EU/EEA increased sharply between 2022 and 2023, when some European countries detected sporadic cases or challenging hospital outbreaks, while others, including Italy, could no longer distinguish such events from regional or national endemicity [9]. Prior studies concerning Italy have independently reported emerging *C. auris* cases [16-19,21,22], but were mostly focused on local contexts, lacking comparative analyses at country level. This study thus explored the *C. auris* genomic landscape in Italy more comprehensively, providing further insight into its clonal diversity and spread.

Leveraging previously established datasets of whole genome sequences from 17 countries [29-32], we accurately delineated major *C. auris* clades (i.e. I to VI). As all Italian isolates belonged to clade I, we conducted in-depth investigations of subclades within this clade (Supplementary Table S1). While no Italian subclade Ia isolates were uncovered, phylogenetic analyses revealed that *C. auris* dissemination in Italy appeared to be associated with a complex population structure, characterised by the emergence and spread of several lineages among subclades Ib and Ic. Interestingly, a genomic comparison with contemporary isolates from Romania also suggested a putative additional subclade (i.e. subclade Id).

Subclade Ic comprised all isolates from the major outbreak detected in Liguria in 2020 (cluster Ob4), as well as a few clonally related isolates from other Italian regions, all characterised by the specific genetic signature *HMG1*^P238H^. In our phylogenetic tree, subclade Ic also included benchmark outbreak clusters from the US (Ob1 from New York; Ob2 from New Jersey; Ob3 from Massachusetts) that were reliably resolved. Study of subclade Ic SNPs confirmed a national Italian epidemic, traceable to the major outbreak in Liguria since 2020, which further spread to Lombardy, Emilia-Romagna and Marche in the earlier phases of *C. auris* emergence in the country [7].

Investigation of subclade Ib identified more recent events with possible imports from southern European countries (i.e. Greece, Albania) and while encompassing fewer Italian cases, this subclade accounted for most of the observed genomic diversity. The majority of Italian subclade Ib isolates were associated with a nosocomial cluster in Lombardy (hospital B, 2022–2025; cluster Ob5) that likely emerged following the transfer of a patient from a Greek hospital (November 2022). Remarkably, the first report of *C. auris* in Lombardy, which occurred within a similar timeframe (October 2022), albeit in a different hospital (hospital A), was also related to an imported case from Greece [18]. Analysis by WGS did not support clonal expansion of a single lineage in Lombardy but rather pointed to multiple independent introductions of *C. auris* in this region. According to the latest ECDC report published in 2025, Greece ranks among EU/EEA countries with the highest cumulative number of *C. auris* cases reported within the 2013–2023 period, reflecting a situation of regional endemicity [9]; moreover, other recent publications have highlighted a rise in *C. auris* fungaemia in Greek intensive-care units (ICUs) [6,34].

Of note, subclade Ib also comprised isolates recovered from patients with proven epidemiological links to Albania (cluster Ob6) but admitted to hospitals from different Italian regions (Liguria, Lombardy, Apulia) and at different times (2023, 2024). Unexpectedly, phylogenetic analysis demonstrated that these isolates shared a high genomic relatedness in terms of separating SNPs and antifungal resistance markers (*TAC1B*^A583S^), consistent with clonal expansion of a single lineage (cluster Ob6). These findings are suggestive of circulation of *C. auris* belonging to subclade Ib in Albania, where no cases have been officially reported to date [9]. Additional investigations are needed to support this hypothesis.

The herein proposed subclade Id included the isolate of a single imported case from Romania recently identified in Tuscany (March 2025). In phylogenetic analysis, this isolate formed a cluster with other isolates collected from different Romanian hospitals during the 2022–2023 period and all isolates within this cluster shared a distinctive pattern of alterations in key antifungal resistance markers (e.g. *TAC1B*^K691E^) and limited separating SNPs. Notably, although Romania first reported *C. auris* cases in early 2022, it figures among the EU/EAA countries experiencing a sharp increase in case numbers during 2023 [9].

Overall, unlike the epidemiological scenario observed in the US, where many of the early *C. auris* cases reflected importation from abroad of different clades [35], early cases in Italy mostly reflected local transmissions following introductions of strains of a single clade [2,3]. Nevertheless, our investigation unveiled an increasing genomic diversity at subclade level, driven by a small number of recent cases in patients likely acquiring *C. auris* through healthcare-associated exposures outside Italy and occasionally giving rise to nosocomial clusters (e.g. cluster Ob5). Consistent with the phylogenomic framework observed in Italy, isolates from India, Pakistan, and the US exhibited phylogenetic mixing across distinct subclades.

According to current international guidelines, several risk factors can guide screening for *C. auris* colonisation, which is advocated for individuals recently admitted to healthcare facilities where transmission is known or suspected; this includes patients coming from national or international areas with documented circulation of *C. auris* or with epidemiological links to other confirmed cases [20,36]. Application of these strategies, however, can be hindered by the lack of up-to-date epidemiological data, including from countries that are not within the ECDC's routine surveillance remit. Furthermore, the ECDC report from September 2025 highlights that the true impact of *C. auris* in the EU/EEA may be underestimated due to the lack of systematic surveillance in many countries there [9]. Considerable heterogeneity moreover exists across Europe in both laboratory capacity and public health measures to control the spread of *C. auris*; as an example, notification of new cases is mandatory in only a few countries, including Italy [9].

In response to the increasing concern that spread of *C. auris* poses, the Italian Ministry of Health issued a national protocol in February 2025 to standardise identification, surveillance, reporting, and containment measures (Circular Letter number 0004265–12/02/2025-DGPRE-DGPRE-P). The document established an active screening strategy to be performed: (i) at hospital admission for individuals with specific risk factors (e.g. prior *C. auris* positivity, healthcare exposure within the previous 12 months, transfer from high-risk units); (ii) weekly in wards with elevated transmission risk (ICUs, surgical and transplant units, oncology/haematology departments); (iii) for patients transferred from high-risk wards, even if initially negative, with follow-up screening for at least 4 weeks or until discharge. In conventional wards, screening was only recommended in the event of detected cases, continuing for 4 weeks after the last case was discharged.

Taken together, present findings underscore the need for stringent screening at inter-facility transfers and in case of patients having a recent history of healthcare exposure abroad, since data concerning *C. auris* spread could be scarce or underestimated due to the limited capacity for surveillance in some countries [9,37].

This study has some limitations, as the established WGS dataset reflects availability of national sequence records rather than systematic sampling, sequencing was primarily performed on outbreak-related isolates, and most recent isolates (from 2023 onwards) from the Ligurian outbreak were not characterised, so that the presence of additional lineages currently circulating in the country cannot be excluded. Furthermore, gaps in genomic surveillance data due to under-documentation in some countries can complicate interpretation of import events. Additional molecular investigations are therefore warranted.

## Conclusions

This study found that the population structure of *C. auris* in Italy reflects both clonal expansion and inter-regional dissemination of subclade Ic, as well as independent introductions from southern Europe of strains of subclade Ib and of a putative subclade Id. We moreover detected some subclade-specific alterations, primarily affecting *TAC1B,* that could potentially serve as molecular markers for tracking certain lineages. With *C. auris* cases also reported in other EU/EEA countries than Italy as well as in some EU enlargement countries, its future sustained control may be challenging. Continued efforts should be directed towards early detection, surveillance and rapid implementation of infection prevention and control measures upon national inter-facility transfers and in case of patients with healthcare exposure abroad, notably from countries where information on *C. auris* circulation is scarce or lacking. In this context, genomic surveillance is key for timely and effective public health responses.

## Data Availability

Raw Illumina reads and draft genomes of *C. auris* isolates sequenced in this study have been deposited in NCBI databases under BioProject no. PRJNA655187.

## SupplementaryData

61000 Supplementary Table S1

## SupplementaryData

480000 Supplementary Figure S1
