# Supplementary material for: Genomic landscape of Candidozyma auris in Italy, 2019 to 2025: emerging diversity of clade I sub-lineages associated with inter-facility transmission and cross-border transfers
Source: Euro Surveill. 2026 May 7;31(18):2500730. doi: 10.2807/1560-7917.ES.2026.31.18.2500730 (PMC13170420; doi:10.2807/1560-7917.ES.2026.31.18.2500730)
Supplement: Supplementary Figure S1 [file 25-00730_SupplementaryFigureS1.pdf]

This supplementary material is hosted by Eurosurveillance as supporting information alongside the article "Genomic landscape of *Candidozyma auris* in Italy, 2019 to 2025: emerging diversity of clade I sub-lineages associated with inter-facility transmission and cross-border transfers", on behalf of the authors, who remain responsible for the accuracy and appropriateness of the content. The same standards for ethics, copyright, attributions and permissions as for the article apply. Supplements are not edited by *Eurosurveillance* and the journal is not responsible for the maintenance of any links or email addresses provided therein

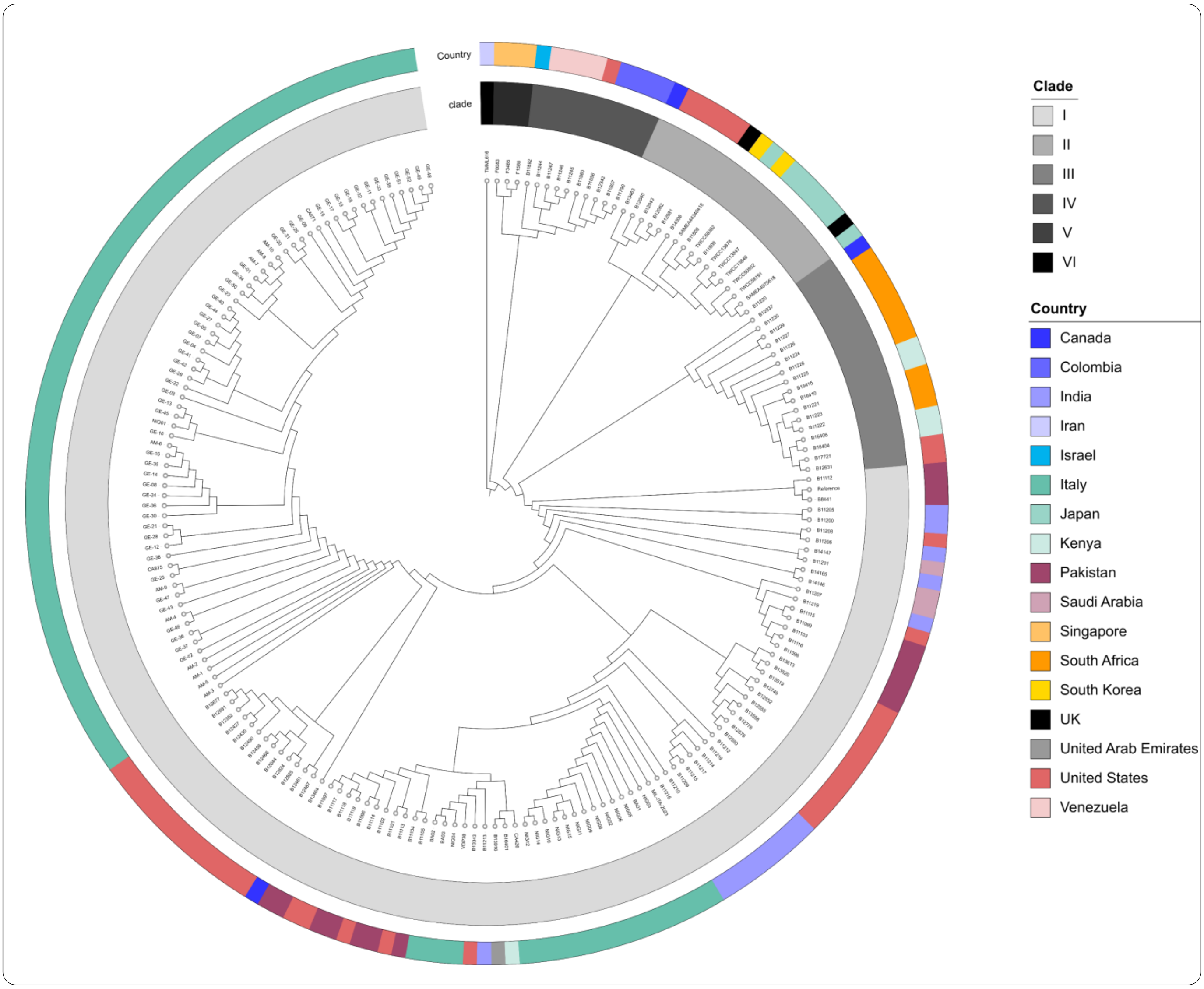

Figure S1. Global phylogenetic analysis of *Candidozyma auris* clustering in the six major phylogenetic clades. Maximum-likelihood phylogenetic tree of *C. auris* genomes characterized in this study (n=85) and representative genomes from different countries and clades (n=112), retrieved from previously established datasets [25-26].
